# Cognitive Disability Among Arab Americans by Nativity Status: Lack of Evidence for the Healthy Migrant Effect

**DOI:** 10.1093/geroni/igaa057.2078

**Published:** 2020-12-16

**Authors:** Tiffany Kindratt, Florence Dallo, Laura Zahodne

**Affiliations:** 1 University of Texas at Arlington, Arlington, Texas, United States; 2 Oakland University, Rochester, Michigan, United States; 3 University of Michigan, Ann Arbor, Michigan, United States

## Abstract

Limited research exists on cognitive disabilities among foreign-born adults, particularly non-Hispanic Arab Americans. We analyzed 10 years (2008-2017) of data from the American Community Survey (ACS) Public Use Microdata Samples (PUMS) (n=5,011,469; ages >45 years). In US-born adults, the age- and sex-adjusted prevalence of cognitive disability among non-Hispanic Arab Americans was 5.3%, which was lower than non-Hispanic whites (6.5%), blacks (10.8%), and Hispanics (10.0%). Among foreign-born adults, the prevalence of cognitive disability was highest, 7.3%, for non-Hispanic Arab Americans compared to all other racial and ethnic groups. Among foreign-born adults, non-Hispanic Arab Americans had 1.24 times greater odds (95% CI=1.12, 1.37) of having a cognitive disability compared to foreign-born non-Hispanic whites. This is the first study to examine cognitive disabilities among US- and foreign-born Arab Americans. More research is needed to better understand factors that may contribute to the increased prevalence of cognitive disabilities for foreign-born adults.

